# Supplementary material for: Two-metal ion mechanism of DNA cleavage by activated, filamentous SgrAI
Source: J Biol Chem. 2024 Jul 14;300(8):107576. doi: 10.1016/j.jbc.2024.107576 (PMC11367474; doi:10.1016/j.jbc.2024.107576)
Supplement: Supporting Information [file mmc1.docx]

**Table S1. Cryo-EM Data and Model Refinement Statistics**

| **EM Data Collection/Processing** | |  |
| --- | --- | --- |
|  | wtSgrAI/40-1/Mg^2^ | K242A/40-1/Mg^2+^ |
| Submission codes | 9BGI, EMD-44513 | 9BGJ, EMD-44514 |
| Microscope | FEI Titan Krios | FEI Titan Krios |
| Voltage (kV) | 300 | 300 |
| Camera | Gatan K2 Summit | K3 quantum |
| Magnification | 165,000 | 105,000 |
| Nominal defocus range (μm) | 0.8-2.0 | 1.0-2.5 |
| Exposure time (s) | 4 | 750 |
| No. of frames | 50 | 50 |
| Frame rate (frames per second) | 12.5 | 66.7 |
| Total fluence (e-/A^2^) | 29.6 | 42.8 |
| Fluence rate (e-/pixel/s) | 5.1 | 39.3 |
| Pixel size (Å) | 0.83 | 0.83 |
| No. of movies | 2,704 | 3,488 |
| Total extracted particles | 821,410 | 2,768,698 |
| No. of particles in final map | 202,664 | 357,489 |
| Symmetry | Helical | Helical |
| Helical parameters (rise, Å / twist, °) | 21.3 / -86.2 | 20.9/-86.2 |
| Resolution, Fourier shell correlation 0.143 (Å) | 3.05 | 3.34 |
| Local resolution range (Å) | 1.78 – 6.06 | 1.79 – 5.30 |
| Directional resolution range from 3D FSC (Å) | 2.89 – 3.16 | 3.12 – 3.59 |
| Sampling Compensation Factor (SCF) | 0.780 | 0.738 |
| Map-sharpening B factor (Å^2^) | 87.9 | 133.2 |
|  |  |  |
| **Atomic model statistics** |  |  |
| Model resolution (Å)  FSC ½ threshold | 3.1 | 3.5 |
| Model composition  Non-hydrogen atoms  Protein residues  Nucleotides  Waters  Ligands (Mg^2+^) | 6511 676 48 177 4 | 6359  672  51  9  2 |
| R.m.s. deviations  Bond lengths (Å)  Bond angles (°) | 0.004 0.518 | 0.003  0.536 |
| Validation  MolProbity score  Clashscore  Poor rotamers (%) | 1.73 4.85  1.77 | 2.43  9.54  4.11 |
| Ramachandran plot  Favored (%)  Allowed (%)  Disallowed (%) | 95.83 4.17  0 | 92.79  7.21  0 |
| CaBLAM outliers (%) | 1.96 | 3.66 |
| Cis proline (%)  Twisted proline (%) Cβ outliers (%) | 0  0  0 | 0  0  0 |
| Average B-factors (min/max/mean, Å^2^)  Protein  Nucleotide  Ligand  Water | 12.9/145.7/54.3  8.8/154.3/52.9  33.4/44.6/39.3  15.7/87.5/40.0 | 31.4/164.8/78.9  29.8/209.2/117.4  46.4/50.4/48.4  28.9/66.25/54.0 |

**Table S2. RMSD between structures of SgrAI bound to DNA***

|  | **Using Both Chains of the SgrAI Dimer** | | | | | **Using a Single Chain of the SgrAI Dimer** | | | | |
| --- | --- | --- | --- | --- | --- | --- | --- | --- | --- | --- |
|  | **SgrAI_WT_/**  **40-1/Mg^2+^** | **SgrAI_K242A_/**  **40-1/Mg^2+^** | **7SS5** | **3DVO** | **3MQY** | **SgrAI_WT_/**  **40-1/Mg^2+^** | **SgrAI_K242A_/**  **40-1/Mg^2+^** | **7SS5** | **3DVO** | **3MQY** |
| **SgrAI_WT_/**  **40-1/Mg^2+^** | - | 0.56  (0.61) | **0.25** (0.30) | 1.90 (1.99) | 1.93 (2.07) | - | 0.47  (0.54) | **0.22** (0.27) | 0.59 (0.70) | 0.68 (0.80) |
| **SgrAI_K242A_/40-1/Mg^2+^** | 0.56 (0.60) | - | 0.56 (0.60) | 1.63 (1.84) | 1.73 (1.92) | 0.47 (0.51) | - | 0.44  (0.48) | 0.54 (0.66) | 0.62 (0.72) |

*Alpha carbons used in RMSD (all atoms in parentheses)

**Table S3. Distances between key atoms in structural superpositions (all atoms of one SgrAI chain used in the superpositions).**

| **Structure 1** | **Structure 2** | **Distance between site A metal ions**  **(Å)** | **Distance between site B metal ions**  **(Å)** | **Distance between P of SP**  **(Å)** | **Distance between O3’**  **(Å)** |
| --- | --- | --- | --- | --- | --- |
| SgrAI_WT_/40-1/Mg^2+^ | SgrAI_WT_/PC/Mg*^2+^*  (PDB ID 6OBJ) | **1.8** | No site B in 6OBJ | No SP in 6OBJ | 1.2 |
| SgrAI_WT_/40-1/Mg^2+^ | SgrAI_WT_/40-1/Ca*^2+^*  (PDB ID 7SS5) | 0.9 | 1.7 | 1.7 | 1.6 |
| SgrAI_WT_/40-1/Mg^2+^ | SgrAI_WT_/18-1/Ca*^2+^*  (PDB ID 3DVO) | 0.8 | No site B in 3DVO | 1.8 | 1.4 |
| SgrAI_WT_/40-1/Mg^2+^ | SgrAI_WT_/18-1/Mg*^2+^*  (PDB ID 3MQY) | 0.7 | 1.2 | 1.7 | **2.2** |
| SgrAI_K242A_/40-1/Mg**^2+^** | SgrAI_WT_/PC/Mg*^2+^*  (PDB ID 6OBJ) | **1.4** | No site B in 6OBJ | No SP in 6OBJ | **0.6** |
| SgrAI_K242A_/40-1/Mg**^2+^** | SgrAI_WT_/40-1/Ca*^2+^*  (PDB ID 7SS5) | **1.6** | 1.9 | 0.8 | 1.6 |
| SgrAI_K242A_/40-1/Mg**^2+^** | SgrAI_WT_/18-1/Ca*^2+^*  (PDB ID 3DVO) | 0.9 | No site B in 3DVO | **0.9** | 1.1 |
| SgrAI_K242A_/40-1/Mg**^2+^** | SgrAI_WT_/18-1/Mg*^2+^*  (PDB ID 3MQY) | 0.7 | 1.1 | **0.8** | **3.3** |
| SgrAI_K242A_/40-1/Mg**^2+^** | SgrAI_WT_/40-1/Mg^2+^ | 0.7 | **1.5** | 1.0 | 1.3 |

**
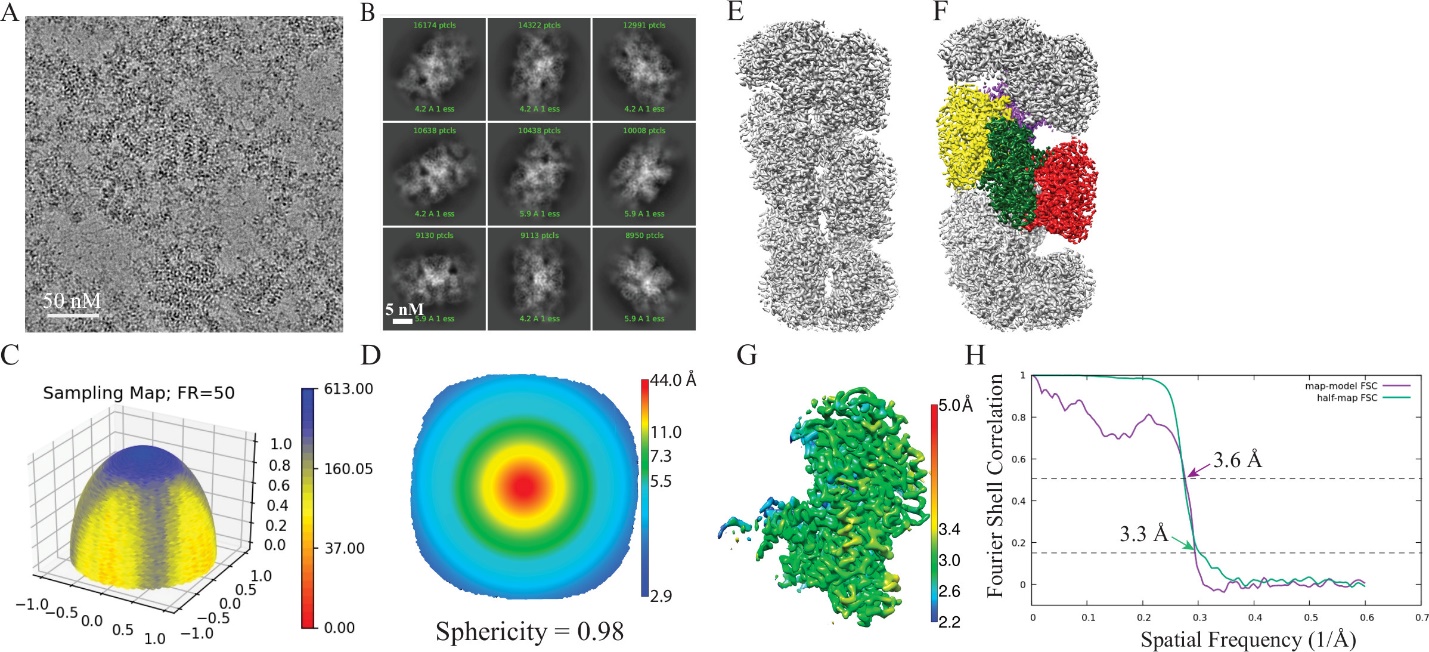
**

**Figure S1. Cryo-EM data and validation of SgrAI_K242A_/40-1/Mg^2+^.** (**A**) A micrograph showing the particle distribution on an ultrAufoil grid overlaid with graphene. (**B**) Example 2D averages from the data used in the final reconstruction map. (**C**) Surface sampling plot derived from the Euler angle distribution, calculated with a Fourier radius set to 50 voxels. The sampling compensation factor (SCF) is indicated in **Table S1** (90,91). (**D**) Central slice through the 3DFSC (89,98) colored by resolution. (**E**) The full cryo-EM map. (**F**) The full cryo-EM map with 4 DBDs colored as in **Fig. 1A**. (**G**) A segmented DBD from the map, colored by local resolution. (**H**) Fourier shell correlation (FSC) curves derived from half-map and map-to-model reconstructions, with FSC cutoffs 0.143 and 0.5 indicated, respectively, as well as nominal resolution values.

**
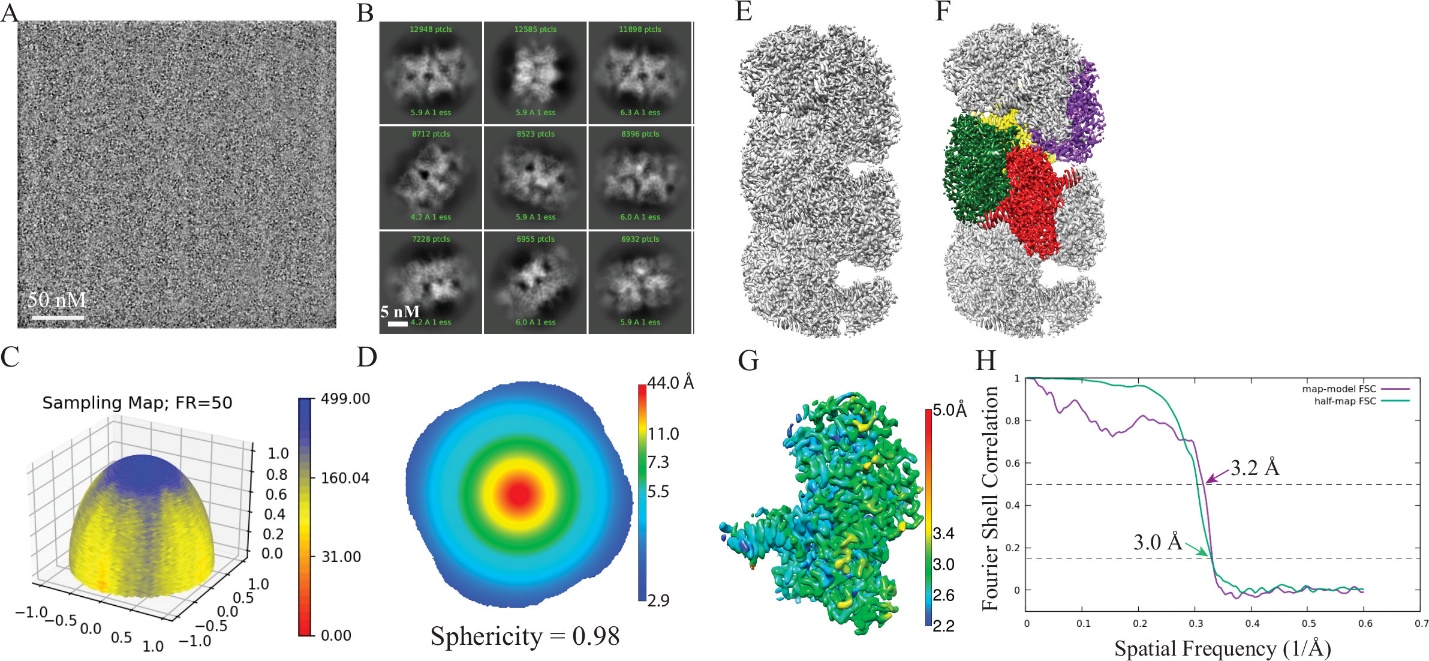
**

**Figure S2. Cryo-EM reconstruction validation of SgrAI_WT_/40-1/Mg^2+^.** (**A**) A micrograph showing the particle distribution on an ultrAufoil grid overlaid with graphene. (**B**) Example 2D averages from the data used in the final reconstruction map. (**C**) Surface sampling plot derived from the Euler angle distribution, calculated with a Fourier radius set to 50 voxels. The sampling compensation factor (SCF) is indicated in **Table S1** (90,91). (**D**) Central slice through the 3DFSC (89,98) colored by resolution. (**E**) The full cryo-EM map. (**F**) The full cryo-EM map with 4 DBDs colored as in **Fig. 1A**. (**G**) A segmented DBD from the map, colored by local resolution. (**H**) Fourier shell correlation (FSC) curves derived from half-map and map-to-model reconstructions, with FSC cutoffs 0.143 and 0.5 indicated, respectively, as well as nominal resolution values.
